# The impact of patient preferences and costs on the appropriateness of spinal manipulation and mobilization for chronic low back pain and chronic neck pain

**DOI:** 10.1186/s12891-019-2904-6

**Published:** 2019-11-07

**Authors:** Patricia M. Herman, Margaret D. Whitley, Gery W. Ryan, Eric L. Hurwitz, Ian D. Coulter

**Affiliations:** 1 RAND Corporation, Santa Monica, CA United States; 2 Office of Public Health Studies, University of Hawaii, Honolulu, HI United States

**Keywords:** Appropriateness of care, Spinal mobilization, Spinal manipulation, Chronic low back pain, Chronic neck pain, Patient preferences, Cost

## Abstract

**Background:**

Although the delivery of appropriate healthcare is an important goal, the definition of what constitutes appropriate care is not always agreed upon. The RAND/UCLA Appropriateness Method is one of the most well-known and used approaches to define care appropriateness from the clinical perspective—i.e., that the expected effectiveness of a treatment exceeds its expected risks. However, patient preferences (the patient perspective) and costs (the healthcare system perspective) are also important determinants of appropriateness and should be considered.

**Methods:**

We examined the impact of including information on patient preferences and cost on expert panel ratings of clinical appropriateness for spinal mobilization and manipulation for chronic low back pain and chronic neck pain.

**Results:**

The majority of panelists thought patient preferences were important to consider in determining appropriateness and that their inclusion could change ratings, and half thought the same about cost. However, few actually changed their appropriateness ratings based on the information presented on patient preferences regarding the use of these therapies and their costs. This could be because the panel received information on average patient preferences for spinal mobilization and manipulation whereas some panelists commented that appropriateness should be determined based on the preferences of individual patients. Also, because these therapies are not expensive, their ratings may not be cost sensitive. The panelists also generally agreed that preferences and costs would only impact their ratings if the therapies were considered clinically appropriate.

**Conclusions:**

This study found that the information presented on patient preferences and costs for spinal mobilization and manipulation had little impact on the rated appropriateness of these therapies for chronic low back pain and chronic neck pain. Although it was generally agreed that patient preferences and costs were important to the appropriateness of M/M for CLBP and CNP, it seems that what would be most important were the preferences of the individual patient, not patients in general, and large cost differentials.

## Background

In the 1990s the RAND Corporation and the University of California, Los Angeles, (UCLA) developed an approach to determine the types of patients for which the use of use of a medical procedure was appropriate: the RAND/UCLA Appropriateness Method (RUAM) [1]. This approach used an expert, mixed clinician- and researcher-based panel to consider the available evidence and then to judge for a particular treatment whether it would be appropriate “for an average group of patients presenting [with this set of clinical indications] to an average US physician who performed the procedure [at the time of the panel determination].” [2] A treatment would be rated as appropriate if: “the expected health benefit (e.g., increased life expectancy, relief of pain, reduction in anxiety, improved functional capacity) exceeds the expected negative consequences (e.g., mortality, morbidity, anxiety, pain, time lost from work) by a sufficiently wide margin that the procedure is worth doing, exclusive of cost.” [2] Panelists were asked to rate the appropriateness of the treatment for patients presenting with hundreds of different clinical scenarios—i.e., all possible clinical presentations.

To date the RUAM has been the most widely used and studied method for defining and identifying clinically appropriate care [3]. The estimates generated by the RUAM have been found to be reliable, [4] reproducible, [5, 6] and valid, [4, 7, 8] with reasonable sensitivity and specificity regarding inappropriate overuse and underuse of healthcare, [9] and predictive of outcomes [8, 10–13].

The RUAM approach to appropriateness makes it feasible to take the best of what is known from research and apply it—using the expertise of experienced clinicians—over the wide range of patients and presentations seen in real-world clinical practice. Clinicians have often been the final translators of evidence into practice, and this approach formalizes the process. A potential major limitation of the RUAM, however, is that it utilizes a definition of appropriateness from a clinical or professional perspective [14, 15]; one that relies solely on safety, efficacy and effectiveness. In contrast, the proceedings of an international World Health Organization workshop stated that while the appropriateness of care is a “complex issue,” [16] across countries: “most definitions of appropriateness address … that care is effective (based on valid evidence); efficient (cost-effectiveness); and consistent with the ethical principles and preferences of the relevant individual, community or society.” [16]^, p2.^

The consideration of including patient preferences and cost-effectiveness in the determination of appropriate care is important given the challenges of patient-centered care and rising healthcare costs. The inclusion of these factors may be especially important for many of the nonpharmacologic interventions, including those encompassed by the term complementary and integrative health (CIH), because of their high need for patient engagement and high rates of self-referral and out-of-pocket costs [17–19].

The RAND Center of Excellence for Research in CIH (CERC) examined the appropriateness of spinal mobilization and manipulation (M/M) for patients with chronic low back pain (CLBP) and chronic neck pain (CNP) [20, 21]. One of the goals of the CERC was to determine the impact of the inclusion of patient preferences and cost effectiveness on appropriateness ratings that were originally made from the clinical perspective. We know of no other study that measured the effect of patient preferences on appropriateness, but did find one other study that examined the impact of considering healthcare resource use on clinical appropriateness ratings [22]. This study found the number of clinical scenarios rated as appropriate for the use of preoperative erythropoietin in elective orthopedic surgery dropped from 66 to 53% when resource constraints (costs) were considered.

This paper presents the results of performing a second round of RUAM appropriateness panels where panelists re-rated the appropriateness of M/M for CLBP and CNP after considering evidence on patients’ preferences for these therapies and the relative cost and cost-effectiveness of M/M compared to other treatment alternatives for CLBP and CNP. We report on whether panelists changed their ratings, and if they did, their statements as to why.

## Methods

The whole CERC study is described in more detail elsewhere [20, 21]. In brief, two panels of expert clinicians and researchers were convened following the traditional RUAM approach [1] in March and May of 2015, for M/M for CLBP and for CNP, respectively [23–25]. In parallel two other CERC projects focused on: 1) capturing data on patient attitudes and preferences for M/M in a large sample using chiropractic care for CLBP and CNP; and 2) the costs and cost-effectiveness of M/M compared to other commonly used interventions for CLBP and CNP. Then in the last year of the CERC (December 2017 for CLBP and January 2018 for CNP), the same appropriateness panels were brought together again; presented with the results of the projects on patient preferences and cost-effectiveness; and asked to re-rate the appropriateness of M/M given the information presented to see if that information changed their ratings.

The methods used to develop the initial ratings of clinical appropriateness followed the traditional RUAM clinical perspective [1] and the resulting ratings are presented in detail in two publicly available RAND Reports [23, 24] and in terms of their impacts on guidelines in another article [25]. In brief, panels were assembled that included content (i.e., research) and clinical experts in CLBP and CNP who did and who did not utilize M/M in their practice. The intent of the selection of the panelists was to balance between clinical experience (for clinical acumen) and content knowledge (to be able to understand the evidence). Studies of the RUAM have also shown that practitioners who perform the therapy are more likely to rate the therapy as appropriate than those who do not, [22, 26–31] thus, we also balanced clinical experience between those who do and do not perform the therapy. That number was large enough to permit diversity of representation while still being small enough to allow everyone to be involved in group discussions. RUAM studies have used panels ranging from 7 to 15 members. It has been found that the reliability of the ratings declines when panel size falls below 6, and improvement in reliability beyond 12 is not substantial [26]. Panelists were each provided with a $1000 honorarium plus travel expenses for their participation, but experience has shown that panelists are honored to be asked and participate willingly and enthusiastically even without enumeration [1].

We used a traditional 9-member panel for CLBP consisting of eight men and one woman. Three panelists were practicing clinicians who used M/M for CLBP in their practice: one chiropractor, one osteopath, and one physical therapist. Three more were practicing clinicians who treat patients with CLBP: one orthopedic surgeon, one internist, and one radiologist. These three panelists were all also content and research experts in CLBP. And three were PhD researchers in CLBP: one chiropractor, and two health services researchers.

Because of the increased attention regarding the safety of cervical manipulation, [32, 33] our CNP panel used a larger 11-member panel consisting of nine men and two women. Four panelists were practicing clinicians who used M/M in their practice: two chiropractors, one osteopath, and one physical therapist. All but the physical therapist were also researchers in CNP. Four panelists were practicing clinicians who treat patients with CNP: one neurologist (who is also a chiropractor, but not practicing as one), one orthopedic surgeon, one internist, and one physiatrist. The first three of these panelists were also CNP researchers. And the last three panelists were all non-practicing chiropractors who were researchers and content experts in CNP. Unfortunately, not as much health services research has been done in CNP, so we utilized other researchers. The same physical therapist served on both the CLBP and CNP panels. Otherwise, panelists were unique to their panel.

Panelists were first presented with a detailed systematic review of the latest evidence on the effectiveness and safety of M/M for CLBP [34] or CNP [35]. Panelists were then asked to rate on a 1–9 scale the extent to which the benefits of each therapy outweigh its risks for each clinical scenario. Ratings of 7–9 (appropriate) were given if the expected health benefit of the treatment exceeded its negative consequences by a sufficiently wide margin that the procedure was worth doing. Ratings of 1–3 (inappropriate) were given if the negative consequences were believed to exceed the treatment’s benefits, and ratings of 4–6 (equivocal) were given if the benefits and negative consequences were roughly equal. Each panelist rated each clinical scenario twice: alone at home and then after seeing other panelists’ unidentified at-home ratings and discussion during an in-person meeting. The clinical scenarios to rate were organized into sections for ease of rating—i.e., once one (the first) clinical scenario in a section was rated, the others only differed by one or two patient characteristics and could be evaluated quickly. The CLBP panel rated the appropriateness of M/M for 900 clinical scenarios (450 assuming that an adequate trial of non-surgical, non-manipulative care of sufficient intensity and duration to normally achieve a favorable response was not tried, and 450 assuming that this course of care was tried and failed) and the CNP panel initially rated 386, but finally rated 372 (186 assuming an adequate trial was not tried, and 186 assuming it was tried and failed). Both panels reported that the at-home ratings took them roughly 2–3 h.

The second round of panels reconvened the same panelists almost 3 years later. Panelists first were provided again with the descriptions of the clinical scenarios and their final ratings from the first (2015) round of panels. They were asked to rerate at home the clinical (effectiveness and safety) appropriateness of M/M for each clinical scenario in response to any new evidence they may have encountered over the years since the first round of panels. Any revisions to panelists’ previous ratings were incorporated into the personalized reports they received at a new all-day in-person meeting (December 2017/January 2018) at RAND offices in Santa Monica, California. These personalized reports showed their ratings in relation to the distribution, but not the identities, of the other panelists’ ratings. Because of the large number of clinical scenarios for CLBP and the similarity in ratings between mobilization and manipulation found in the initial ratings, [25] the CLBP appropriateness panel only re-rated the appropriateness of spinal manipulation. The CNP appropriateness panel re-rated all clinical scenarios for CNP for both spinal mobilization and manipulation. Panelists were again each provided with a $1000 honorarium plus travel expenses for their participation.

At the in-person meeting the panelists were given information and presentations based on the results of the other CERC study projects. One presentation was on the expectations and preferences of a large sample of patients who use chiropractic care for their CLBP and CNP [36]. The main points made here were that: 1) about two-thirds of patients were not seeking a cure for their pain; instead, they were seeking temporary relief or prevention of the pain returning [37]; 2) these patients had a strong preference for chiropractic care (i.e., spinal mobilization and manipulation) and the majority of patients said that avoiding surgery (84%) and avoiding prescription medicine (75%) were very or extremely important to their decision to use chiropractic care [36]; and 3) chiropractic care was not the only coping mechanism patients were using to manage their pain; it was one element in their overall coping strategy.

The second presentation was on the relative costs and cost-effectiveness of M/M as compared to other common nonsurgical interventions for CLBP and CNP from the health system and societal perspectives. M/M was shown to be mid-level in terms of upfront intervention costs with home and group interventions such as exercise and yoga being somewhat less expensive and more intensive interventions such as injections being more expensive. In terms of cost-effectiveness, the interventions examined for CLBP tended to be more effective and cost-effective than the interventions for CNP. For both conditions M/M tended to be roughly of similar effectiveness and cost-effectiveness as the other interventions studied, and for CLBP M/M tended to be cost neutral (payer perspective) or cost saving (societal perspective).

After these presentations, the panelists were asked to re-rate the appropriateness of spinal manipulation for CLBP and M/M for CNP taking the information presented into consideration to the extent they thought applicable. Panelists were also asked to write notes describing whether they thought information on preferences and costs were important to the determination of appropriateness and the circumstances under which this information would change their ratings even if they did not change their ratings in this round.

### Analysis

We examined the results both in terms of the numbers of individual panelists who made changes and the number of individual ratings changed between four sets of ratings: 1) final ratings from the 2015 panels compared to new at-home ratings (an indication of the stability of appropriateness ratings over time); 2) at-home ratings compared to ratings made during the in-person meeting that were not due to the information presented on preferences and cost (additional information on the stability of ratings even after further discussions and consideration); and 3) ratings not due to the presentations compared to those reported as being due to the presentations (an indication of the impact on appropriateness of the information presented on preferences and costs).

For each new set of ratings, we capture statistics on the number of panelists who changed ratings, and the number of clinical scenarios for which ratings were changed. We then calculate across all clinical scenarios for each set of new ratings the average median rating, the dispersion of the ratings measured by the mean absolute deviation (MAD) from the median, the proportions of clinical scenarios for which there was agreement and disagreement across panelists, and the proportions of clinical scenarios rated as appropriate, equivocal and inappropriate.

For a classic 9-member panel, agreement for a clinical scenario was defined by having at least 7 of the ratings in any of the 3-point regions of the scale, and disagreement was defined as having at least three panelists’ ratings in the 1–3 range and at least three in the 7–9 range. For an 11-member panel disagreement was defined as having at least four panelists’ ratings in each the 1–3 and 7–9 ranges. If there was no disagreement and the median value of the ratings across the panel is 1–3, then the therapy was rated as inappropriate for that clinical scenario. If there is no disagreement and the median value of the ratings is 7–9, the therapy was rated as appropriate. The appropriateness for a therapy for a clinical scenario was rated as equivocal if: 1) most panelists gave a rating of 4, 5 or 6—i.e., most agreed that benefits generally equaled risks; 2) panelists gave widely polarized ratings—i.e., there was disagreement; or 3) panelists’ ratings were scattered across the scale—i.e., there was substantial uncertainty as to appropriateness—and the median value was in the 4–6 range.

Paired t tests were used to compare average median ratings and χ^2^ tests were used to compare frequencies of agreement and disagreement and appropriate and inappropriate ratings across the four sets of ratings. Correlation coefficients were calculated to compare the final ratings from the 2015 panels and the final ratings from these rounds for each clinical scenario for each panelist and across all panelists.

Panelists’ notes about whether and how preferences and costs could change appropriateness ratings were analyzed using an inductive process. Two researchers reviewed and discussed the written comments and created a list of emergent themes.

## Results

Table 1 gives an overview of the results. Only 2 CLBP and 4 CNP panelists changed ratings at home, and although they collectively changed the ratings for hundreds of clinical scenarios, these only represented 4% (CLBP) and 3% (CNP) of all ratings. More panelists made changes during the in-person meetings, but few panelists made changes to their ratings based on the results of the presentations on preferences and costs. Instead, the ratings were remarkably stable even after adding the information from the presentations. Across all panelists the correlation between the final individual ratings of the 2015 panels and the final individual ratings from these panels was 0.97 with individual panelists’ correlations ranging from 0.88 to 1.00. The lower part of Table 1 indicates that half to almost 90% of panelists believed that it was possible that other information on preferences and/or costs could cause them to change their ratings. We review the comments made by panelists regarding the circumstances under which they could change their ratings below.

**Table 1:** Characteristics and Results of Appropriateness Panels

	Chronic Low Back Pain		Chronic Neck Pain	
	Panelists^b^	Clinical Scenarios^a,b^	Panelists	Clinical Scenarios^a^
Total numbers of panelists and clinical scenarios rated across panelists	9 (100%)	8100 (100%)	11 (100%)	8184 (100%)
Changed ratings at home	2 (22%)	335 (4%)	4 (36%)	281 (3%)
Changed ratings during in-person meeting	5 (63%)	219 (3%)	10 (91%)	758 (9%)
Changed ratings because of preferences	1 (13%)	8 (0%)	5 (45%)	162 (2%)
Changed ratings because of costs	1 (13%)	3 (0%)	0 (0%)	0 (0%)
Panelists who said ratings could be changed because of preferences	7 (88%)		8 (73%)	
Panelists who said ratings could be changed because of costs	4 (50%)		6 (55%)	

^a^The denominator for the number of clinical scenarios where ratings were changed is the number of panelists times the number of clinical scenarios, so 9*900 or 8100 for chronic low back pain and 11*744 or 8184 for chronic neck pain

^b^One chronic low back pain panelist could not attend the in-person meeting; therefore, the chronic low back pain percentages for the in-person meeting and below are calculated over 8 panelists and 7200 (8*900) ratings

Tables 2-4 provide more detail on the changes made at home and during the in-person meeting for the CLBP panel for spinal manipulation, the CNP panel for spinal mobilization, and the CNP panel for spinal manipulation, respectively. As indicated by the statistics shown in Table 1, very little changed between the final 2015 ratings using the traditional RUAM method and the final ratings for this second set of appropriateness panels. The main changes seen in Table 2 (CLBP spinal manipulation) were that appropriateness ratings decreased slightly and agreement generally doubled between the at-home and in-person ratings not due to the presentations.

**Table 2:** Changes Made in the Chronic Low Back Pain Panel for Spinal Manipulation

	No other adequate conservative care				Nonmanipulative conservative care has failed			
	Final ratings following traditional RUAM	At home ratings	In-person ratings not due to presentations	In-person ratings due to presentations	Final ratings following traditional RUAM	At home ratings	In-person ratings not due to presentations	In-person ratings due to presentations
Average median (1–9 scale)	5.2	5.2	5.0	5.0	5.4	5.4	5.2	5.2
Average MAD from median	1.0	1.0	0.9	0.9	1.0	1.0	0.9	0.9
Agreement [n (%)]*	80 (17.8%)	79 (17.6%)	177 (39.3%)	177 (39.3%)	107 (23.8%)	104 (23.1%)	197 (43.8%)	198 (44.0%)
Uncertain [n (%)]	369 (82.0%)	370 (82.2%)	272 (60.4%)	272 (60.4%)	343 (76.2%)	346 (76.9%)	253 (56.2%)	252 (56.0%)
Disagreement [n (%)]	1 (0.2%)	1 (0.2%)	1 (0.2%)	1 (0.2%)	0 (0.0%)	0 (0.0%)	0 (0.0%)	0 (0.0%)
Inappropriate	58 (12.9%)	57 (12.7%)	60 (13.3%)	60 (13.3%)	48 (10.7%)	47 (10.4%)	50 (11.1%)	50 (11.1%)
Equivocal	318 (70.7%)	318 (70.7%)	314 (69.8%)	314 (69.8%)	303 (67.3%)	304 (67.6%)	302 (67.1%)	302 (67.1%)
Agreement and equivocal	23 (16.2%)	22 (4.9%)	118 (26.2%)	118 (26.2%)	43 (9.6%)	42 (9.3%)	134 (29.8%)	134 (29.8%)
Disagreement	1 (0.2%)	1 (0.2%)	1 (0.2%)	1 (0.2%)	0 (0.0%)	0 (0.0%)	0 (0.0%)	0 (0.0%)
Uncertain and equivocal	294 (56.4%)	295 (65.6%)	195 (43.3%)	195 (43.3%)	260 (57.8%)	262 (58.2%)	168 (37.3%)	168 (37.3%)
Appropriate	74 (16.4%)	75 (16.7%)	76 (16.9%)	76 (16.9%)	99 (22.0%)	99 (22.0%)	98 (21.8%)	98 (21.8%)
Total	450	450	450	450	450	450	450	450

RUAM = RAND/UCLA Appropriateness Method – the traditional version of this did not consider patient preferences and cost

*The numbers of clinical scenarios for which there was agreement across panelists increased significantly (*p* < .001) between at-home and in-person ratings under both conditions

**Table 3:** Changes Made in the Chronic Neck Pain Panel for Spinal Mobilization

	No other adequate conservative care				Nonmanipulative conservative care has failed			
	Final ratings following traditional RUAM	At home ratings	In-person ratings not due to presentations	In-person ratings due to presentations	Final ratings following traditional RUAM	At home ratings	In-person ratings not due to presentations	In-person ratings due to presentations
Average median (1–9 scale)	4.3	4.3	4.3	4.2	4.7	4.7	4.6	4.6
Average MAD from median	1.2	1.3	1.1	1.1	1.2	1.1	1.0	1.0
Agreement [n (%)]*	70 (37.6%)	65 (34.9%)	76 (40.9%)	78 (41.9%)	82 (44.1%)	81 (43.5%)	91 (48.9%)	92 (49.5%)
Uncertain [n (%)]	114 (61.3%)	119 (64.0%)	110 (59.1%)	108 (58.1%)	100 (53.8%)	101 (54.3%)	95 (51.1%)	94 (50.5%)
Disagreement [n (%)]	2 (1.1%)	2 (1.1%)	0 (0.0%)	0 (0.0%)	4 (2.2%)	4 (2.2%)	0 (0.0%)	0 (0.0%)
Inappropriate	64 (34.4%)	65 (34.9%)	67 (36.0%)	69 (37.1%)	51 (10.0%)	51 (27.4%)	58 (31.2%)	58 (31.2%)
Equivocal	95 (51.1%)	95 (51.1%)	94 (50.5%)	92 (49.5%)	97 (65.8%)	96 (51.6%)	90 (48.4%)	90 (48.4%)
Agreement and equivocal	10 (5.4%)	7 (3.8%)	14 (7.5%)	13 (7.0%)	19 (10.2%)	19 (10.2%)	24 (12.9%)	24 (12.9%)
Disagreement	2 (1.1%)	2 (1.1%)	0 (0.0%)	0 (0.0%)	4 (2.2%)	4 (2.2%)	0 (0.0%)	0 (0.0%)
Uncertain and equivocal	83 (44.6%)	86 (46.2%)	80 (43.0%)	79 (42.5%)	74 (39.8%)	73 (39.2%)	66 (35.5%)	66 (35.5%)
Appropriate	27 (14.5%)	26 (14.0%)	25 (13.4%)	25 (13.4%)	38 (20.4%)	39 (21.0%)	38 (20.4%)	38 (20.4%)
Total	186	186	186	186	186	186	186	186

RUAM = RAND/UCLA Appropriateness Method – the traditional version of this did not consider patient preferences and cost

*The numbers of clinical scenarios for which there was agreement across panelists increased significantly between at-home and in-person ratings; p < .001 for when there was no other adequate conservative care and *p* = .0076 for when nonmanipulative conservative care has failed

**Table 4:** Changes Made in the Chronic Neck Pain Panel for Spinal Manipulation

	No other adequate conservative care				Nonmanipulative conservative care has failed			
	Final ratings following traditional RUAM	At home ratings	In-person ratings not due to presentations	In-person ratings due to presentations	Final ratings following traditional RUAM	At home ratings	In-person ratings not due to presentations	In-person ratings due to presentations
Average median (1–9 scale)	3.9	3.8	3.8	3.8	4.2	4.2	4.2	4.2
Average MAD from median	1.3	1.3	1.2	1.2	1.2	1.2	1.2	1.2
Agreement [n (%)]*	63 (33.9%)	61 (32.8%)	74 (39.8%)	75 (40.3%)	75 (40.3%)	72 (38.7%)	84 (45.2%)	85 (45.7%)
Uncertain [n (%)]	122 (65.6%)	124 (66.7%)	111 (59.7%)	110 (59.1%)	110 (59.1%)	113 (60.8%)	102 (54.8%)	101 (54.3%)
Disagreement [n (%)]	1 (0.5%)	1 (0.5%)	1 (0.5%)	1 (0.5%)	1 (0.5%)	1 (0.5%)	0 (0.0%)	0 (0.0%)
Inappropriate	80 (43.0%)	82 (44.1%)	83 (44.6%)	84 (45.2%)	66 (35.5%)	67 (36.0%)	66 (35.5%)	67 (36.0%)
Equivocal	90 (48.4%)	89 (47.8%)	88 (47.3%)	87 (46.8%)	94 (50.5%)	91 (48.9%)	92 (49.5%)	92 (49.5%)
Agreement and equivocal	4 (2.2%)	4 (2.2%)	10 (5.4%)	10 (5.4%)	7 (3.8%)	7 (3.8%)	12 (6.5%)	12 (6.5%)
Disagreement	1 (0.5%)	1 (0.5%)	1 (0.5%)	1 (0.5%)	1 (0.5%)	1 (0.5%)	0 (0.0%)	0 (0.0%)
Uncertain and equivocal	85 (45.7%)	84 (45.2%)	77 (41.4%)	76 (40.9%)	86 (46.2%)	83 (44.6%)	80 (43.0%)	80 (43.0%)
Appropriate	16 (8.6%)	15 (8.1%)	15 (8.1%)	15 (8.1%)	26 (14.0%)	28 (15.1%)	28 (15.1%)	27 (14.5%)
Total	186	186	186	186	186	186	186	186

RUAM = RAND/UCLA Appropriateness Method – the traditional version of this did not consider patient preferences and cost

*The numbers of clinical scenarios for which there was agreement across panelists increased significantly (p < .001) between at-home and in-person ratings under both conditions

In secondary analyses we discovered that the reduction in average median appropriateness ratings and corresponding increase in clinical scenarios rated as inappropriate were solely due to one panelist who could not attend the in-person meeting. That panelist consistently gave higher (more appropriate) ratings to all scenarios and his/her absence lowered the resulting in-person scores. Even accounting for the absence of this panelist, there was a real (*p* < .001) increase in the amount of agreement (and a corresponding decrease in the number of scenarios rated as uncertain) across panelists once they met in-person. Tables 3 and 4 show smaller, but still significant (*p*-values from <.001 to .008) increases in agreement between the at-home and in-person ratings for CNP spinal mobilization and manipulation.

The changes between the last two columns (i.e., between “in-person ratings not due to presentations” and “in-person ratings due to presentations”) in each set in Tables 2-4 show the changes due to the presentations on preferences and costs. Although Table 1 indicated that a few CLBP panelists changed a few ratings because of these presentations, these few rating changes did not result in any changes to average median ratings or to the number of clinical scenarios rated as appropriate, equivocal and inappropriate in Table 2.

On the other hand, more panelists changed more ratings in the CNP panel, especially in response to information on patient preferences (Table 1), and these did translate into minor changes between the last two columns in each set in Tables 3 and 4—generally a bit more agreement across panelists, and a few more clinical scenarios rated as inappropriate. In all cases between 71 and 75% of the ratings changes were made by two panelists. The rating changes of the first of these panelists had the largest impact on appropriateness. This panelist consistently lowered his/her ratings citing the belief that poor expectations on the part of the patient increased the risk for poor outcomes. The clinical scenarios where these rating reductions resulted in a change in status from equivocal to inappropriate were associated with the patient having no response to prior manipulative treatment, which may have been believed to affect those patients’ expectations. The other panelist consistently increased their ratings, especially for scenarios where the patient was under continued psychosocial stress citing the belief that the provider would take the patient’s psychosocial factors into account (e.g., low recovery expectations, activity avoidance) and address them as part of management.

We captured comments from most panelists regarding the circumstances under which they thought that preferences and costs could change their ratings. Regarding patient expectations and preferences, almost three-quarters of CNP panelists and all but one CLBP panelist said that these were important to appropriateness. Several panelists commented that patient expectations and preferences should not be considered if the therapy was otherwise rated as inappropriate for that clinical scenario, and one said that they should only be considered if the choice doesn’t affect costs to the healthcare system. Several others also stated that preferences could affect expectations, which in turn affect outcomes, and that while all preferences are important, most relevant here are individual patients’ preferences for the risks and benefits of a given treatment over reasonable alternatives (e.g., not just that one therapy would be more enjoyable).

Half of panelists thought that cost was important to appropriateness, especially if there was a large cost differential compared to alternatives and if the therapy was not otherwise inappropriate. Some of these panelists thought that only the cost to the patient should be considered, and others focused on the cost to the healthcare system while acknowledging that those costs can differ across settings.

## Discussion

We reassembled panels of content and clinical experts to reconsider their ratings of the appropriateness of spinal mobilization and manipulation for CLBP and CNP clinical scenarios after receiving data on patient preferences and costs. Most panelists who changed their ratings did so at home after thinking about the scenarios again or during the in-person meeting after conversations with fellow panelists. According to their self-report, only a few panelists changed a few of their ratings because of the information presented regarding preferences and costs. Nevertheless, a larger number of panelists said that this information was important to the appropriateness of a therapy and could change their ratings under certain circumstances. Information on patient preferences (and especially through their impact on expectations and outcomes), and costs to patients and to the health care system could change appropriateness ratings, but only for clinical scenarios not otherwise judged to be clinically inappropriate. In this last point, the panelists seemed to be indicating what the Dartmouth Atlas calls preference-sensitive care [38].

Adding individual patient preferences and considering costs to the patient in addition to clinical (effectiveness and safety) appropriateness can be considered as defining appropriateness from the patient perspective [3, 14]. In contrast, adding broader resource use and health system cost-effectiveness to appropriateness can be seen as representing the population, health system, and/or societal perspective [3, 14, 15]. From their comments it seems that although some panelists recognized the need for the broader health system or societal perspective, most saw the value of considering the patient perspective of appropriateness. A recent review on the concept of appropriateness found definitions that included one or more of five main categories: evidence-based care, clinical expertise, patient centered-ness, resource use, and equity [15]. The traditional RUAM can be seen as including the first two (evidence-based care and clinical expertise) within clinical appropriateness, and in this project we considered the addition of the next two (patient centered-ness and resource use).

In this study we brought panelists back more than 2.5 years later and found that overall their new ratings correlated 0.97, and individual panelists’ new ratings correlated 0.88 to 1.00, with their final ratings in 2015. Another study asked panelists to rerate a sample of clinical scenarios after a period of 6 to 8 months had elapsed and found correlations between ratings of 0.75 to 0.96 across panelists [4]. It is unclear but likely that this older study had panelists rerate from scratch, whereas our panelists rerated after seeing their previous ratings. In any case, these panel ratings seem to be quite stable over time.

One other study looked at the impact on ratings of clinical appropriateness when healthcare resource use was included in the deliberations [22]. They found that the percent of clinical scenarios rated as appropriate dropped from 66 to 53% when resource constraints were considered. We did not see any change in ratings due to the information presented on the costs of M/M for CLBP and CNP, but M/M is relatively inexpensive and well within the range of costs shown by treatment alternatives. The study that found the reduction in ratings of appropriateness when costs were considered was of the use of preoperative erythropoietin in elective orthopedic surgery and erythropoietin is expensive. In any case, half of the panelists indicated that cost could affect appropriateness under certain circumstances.

This study benefits from the reassembly of panels of clinical and content experts well-versed in the clinical appropriateness method and from their application of this method with the addition of information on patient preferences and costs across hundreds of clinical scenarios. However, these benefits could also be the source of study weaknesses. It could be that the effort involved in learning this technique and applying it across so many clinical scenarios prevented panelists from changing appropriateness ratings in response to new information beyond efficacy, effectiveness and safety. The difference between the small number of panelists who changed ratings and the larger number of panelists who said that more information presented on preferences and costs could change ratings indicates that the information we presented was not in itself compelling enough to change ratings. Preferences were measured in a population already using spinal mobilization and manipulation and presented as population averages, and these therapies have mid-range patient and healthcare system costs compared to alternatives.

## Conclusions

This study found that information on patient preferences and costs for spinal mobilization and manipulation had little impact on the rated appropriateness of these therapies for chronic low back pain and chronic neck pain. The majority of panelists agreed that patient preferences could make a difference especially as preferences can affect expectations and outcomes. However, the information presented to the panel on measured patient preferences for M/M only resulted in a few changes to final appropriateness ratings. Half of panelists said that costs could make a difference to appropriateness, but M/M is not expensive and is mid-range among alternatives for cost-effectiveness. Therefore, few panelists changed their ratings. Although it was generally agreed that patient preferences and costs were important to the appropriateness of M/M for CLBP and CNP, it seems that the preferences of the individual patient, not patients in general, and large cost differentials would make the most difference in the appropriateness of this care.

## Data Availability

The datasets used and/or analysed during the current study are available from the corresponding author on reasonable request.

## Abbreviations

CERC: RAND Center of Excellence for Research in CIH
CIH: Complementary and integrative health
CLBP: Chronic low-back pain
CNP: Chronic neck pain
M/M: Spinal mobilization and manipulation
MAD: Mean absolute deviation
RUAM: RAND/UCLA Appropriateness Method
UCLA: University of California, Los Angeles

## References

[1] Fitch K, Bernstein SJ, Aguilar MD, Burnand B, LaCalle JR, Lazaro P (2001). RAND/UCLA appropriateness method User's manual.

[2] Brook RH, Chassin MR, Fink A, Solomon DH, Kosecoff J, Park RE (1986). A method for the detailed assessment of the appropriateness of medical technologies. Int J Technol Assess Health Care.

[3] Sanmartin C, Murphy K, Choptain N, Conner-Spady B, McLaren L, Bohm E (2008). Appropriateness of healthcare interventions: concepts and scoping of the published literature. Int J Technol Assess Health Care.

[4] Merrick NJ, Fink A, Park RE, Brook RH, Kosecoff J, Chassin MR (1987). Derivation of clinical indications for carotid endarterectomy by an expert panel. Am J Public Health.

[5] Shekelle PG, Kahan JP, Bernstein SJ, Leape LL, Kamberg CJ, Park RE (1998). The reproducibility of a method to identify the overuse and underuse of medical procedures. New Engl J Med.

[6] Tobacman JK, Scott IU, Cyphert S, Zimmerman B (1999). Reproducibility of measures of overuse of cataract surgery by three physician panels. Med Care.

[7] Kahn Katherine L., Park R E, Vennes Jack, Brook Robert H. (1992). Assigning Appropriateness Ratings for Diagnostic Upper Gastrointestinal Endoscopy Using Two Different Approaches. Medical Care.

[8] Shekelle PG, Chassin MR, Park RE (1998). Assessing the predictive validity of the RAND/UCLA appropriateness method criteria for performing carotid endarterectomy. Int J Technol Assess Health Care.

[9] Shekelle PG, Park RE, Kahan JP, Leape LL, Kamberg CJ, Bernstein SJ (2001). Sensitivity and specificity of the RAND/UCLA appropriateness method to identify the overuse and underuse of coronary revascularization and hysterectomy. J Clin Epidemiol.

[10] Selby JV, Fireman BH, Lundstrom RJ, Swain BE, Truman AF, Wong CC (1996). Variation among hospitals in coronary-angiography practices and outcomes after myocardial infarction in a large health maintenance organization. New Engl J Med.

[11] Normand S-LT, Landrum MB, Guadagnoli E, Ayanian JZ, Ryan TJ, Cleary PD (2001). Validating recommendations for coronary angiography following acute myocardial infarction in the elderly: a matched analysis using propensity scores. J Clin Epidemiol.

[12] Kravitz RL, Laouri M, Kahan JP, Guzy P, Sherman T, Hilborne L (1995). Validity of criteria used for detecting underuse of coronary revascularization. JAMA.

[13] Hemingway H, Crook AM, Feder G, Banerjee S, Dawson JR, Magee P (2001). Underuse of coronary revascularization procedures in patients considered appropriate candidates for revascularization. New Engl J Med.

[14] Hopkins A, Fitzpatrick R, Foster A (1993). What do we mean by appropriate health care. Qual Health Care.

[15] Robertson-Preidler J, Biller-Andorno N, Johnson TJ (2017). What is appropriate care? An integrative review of emerging themes in the literature. BMC Health Serv Res.

[16] World Health Organization, Regional Office for Europe (2000). Appropriateness in Health Care Services: Report on a WHO Workshop.

[17] Barnes PM, Bloom B, Nahin RL, Stussman BJ (2009). Costs of complementary and alternative medicine (CAM) and frequency of visits to CAM practitioners, United States, 2007.

[18] Heyward J, Jones CM, Compton WM, Lin DH, Losby JL, Murimi IB (2018). Coverage of nonpharmacologic treatments for low back pain among US public and private insurers. JAMA Netw Open.

[19] Eisenberg DM, Davis RB, Ettner SL, Appel S, Wilkey S, Van Rompay M (1998). Trends in alternative medicine use in the United States, 1990-1997: results of a follow-up national survey. JAMA.

[20] Coulter ID, Herman PM, Ryan GW, Hays RD, Hilton LG, Whitley MD (2019). Researching the appropriateness of care in the complementary and integrative health (CIH) professions: part 1. J Manip Physiol Ther.

[21] Coulter ID, Herman PM, Ryan GW, Hays RD, Hilton LJ, Team CERC (2019). The challenge of determining appropriate Care in the era of patient-centered care and rising health care costs. J Health Serv Res Policy.

[22] Taffé P, Burnand B, Wietlisbach V, Vader J-P (2004). Influence of clinical and economical factors on the expert rating of appropriateness of preoperative use of recombinant erythropoietin in elective orthopedic surgery patients. Med Decis Mak.

[23] Coulter ID, Whitley MD, Hurwitz EL, Vernon H, Shekelle PG, Herman PM (2018). Determining the Appropriateness of Spinal Manipulation and Mobilization for Chronic Low Back Pain. Indications and Ratings by a Multidisciplinary Expert Panel.

[24] Coulter ID, Whitley MD, Vernon H, Hurwitz EL, Shekelle PG, Herman PM (2018). Determining the Appropriateness of Spinal Manipulation and Mobilization for Chronic Neck Pain. Indications and Ratings by a Multidisciplinary Expert Panel.

[25] Herman PM, Hurwitz EL, Shekelle PG, Whitley MD, Coulter ID (2019). Clinical scenarios for which spinal mobilization and manipulation are considered by an expert panel to be inappropriate (and appropriate) for patients with chronic low Back pain. Med Care.

[26] Kahan JP, Park RE, Leape LL, Bernstein SJ, Hilborne LH, Parker L (1996). Variations by specialty in physician ratings of the appropriateness and necessity of indications for procedures. Med Care.

[27] Leape LL, Park RE, Kahan JP, Brook RH (1992). Group judgments of appropriateness: the effect of panel composition. Int J Qual Health Care.

[28] Coulter I, Adams A, Shekelle P (1995). Impact of varying panel membership on ratings of appropriateness in consensus panels: a comparison of a multi-and single disciplinary panel. Health Serv Res.

[29] Ayanian JZ, Landrum MB, Normand S-LT, Guadagnoli E, McNeil BJ (1998). Rating the appropriateness of coronary angiography—do practicing physicians agree with an expert panel and with each other?. New Engl J Med.

[30] Fraser GM, Pilpel D, Kosecoff J, Brook RH (1994). Effect of panel composition on appropriateness ratings. Int J Qual Health Care.

[31] FITCH K, LÁZARO P, AGUILAR MD, MARTÍN Y, BERNSTEIN SJ (1999). Physician recommendations for coronary revascularization: variations by clinical speciality. Eur J Pub Health.

[32] Cassidy JD, Boyle E, Côté P, Hogg-Johnson S, Bondy SJ, Haldeman S (2017). Risk of carotid stroke after chiropractic care: a population-based case-crossover study. J Stroke Cerebrovasc Dis.

[33] Nielsen SM, Tarp S, Christensen R, Bliddal H, Klokker L, Henriksen M (2017). The risk associated with spinal manipulation: an overview of reviews. Syst Rev.

[34] Coulter ID, Crawford C, Hurwitz EL, Vernon H, Khorsan R, Booth MS (2018). Manipulation and mobilization for treating chronic low back pain: a systematic review and meta-analysis. Spine J.

[35] Coulter ID, Crawford C, Hurwitz EL, Vernon H, Khorsan R, Booth MS (2019). Manipulation and mobilization for treating chronic nonspecific neck pain: a systematic review and meta-analysis. Pain Physician.

[36] Herman PM, Kommareddi M, Sorbero ME, Rutter CM, Hays RD, Hilton LG (2018). Characteristics of chiropractic patients being treated for chronic low back and chronic neck pain. J Manip Physiol Ther.

[37] Herman Patricia M., Edgington Sarah E., Ryan Gery W., Coulter Ian D. (2019). Prevalence and Characteristics of Chronic Spinal Pain Patients with Different Hopes (Treatment Goals) for Ongoing Chiropractic Care. The Journal of Alternative and Complementary Medicine.

[38] Center for the Evaluative Clinical Sciences (2007). Preference-Sensitive Care. A Dartmouth Atlas Project Topic Brief.

